# Microsporidian Parasites Found in the Hemolymph of Four Baikalian Endemic Amphipods

**DOI:** 10.1371/journal.pone.0130311

**Published:** 2015-06-18

**Authors:** Ekaterina V. Madyarova, Renat V. Adelshin, Mariya D. Dimova, Denis V. Axenov-Gribanov, Yulia A. Lubyaga, Maxim A. Timofeyev

**Affiliations:** 1 Irkutsk State University, Institute of Biology, Irkutsk, Russia; 2 Irkutsk Anti-Plague Research Institute of Siberia and Far East, Irkutsk, Russia; Institute of Plant Physiology and Ecology, CHINA

## Abstract

At present, approximately 187 genera and over 1300 species of Microsporidia have been described, among which almost half infect aquatic species and approximately 50 genera potentially infect aquatic arthropods. Lake Baikal is the deepest and one of the oldest lakes in the world, and it has a rich endemic fauna with a predominance of arthropods. Among the arthropods living in this lake, amphipods (*Crustacea*) are the most dominant group and are represented by more than 350 endemic species. Baikalian amphipods inhabit almost all depths and all types of substrates. The age and geographical isolation of this group creates excellent opportunities for studying the diversity, evolution and genetics of host-parasite relationships. However, despite more than 150 years of study, data investigating the microsporidia of Lake Baikal remain incomplete. In this study, we used molecular genetic analyses to detect microsporidia in the hemolymph of several endemic species of amphipods from Lake Baikal. We provide the first evidence that microsporidian species belonging to three genera (*Microsporidium*, *Dictyocoela* and *Nosema*) are present in the hemolymph of Baikalian endemic amphipods. In the hemolymph of *Eulimnogammarus verrucosus*, we detected SSU rDNA of microsporidia belonging to the genus Nozema. In the hemolymph of *Pallasea cancellous*, we found the DNA of *Microsporidium sp*. similar to that in other Baikalian endemic amphipods; *Dictyocoela sp*. was found in the hemolymph of *Eulimnogammarus marituji* and *Acanthogammarus lappaceus longispinus*.

## Introduction

Microsporidia are ancient eukaryotic intracellular parasites. Their life cycles include multiple stages, they can change hosts and they affect all eukaryotic organisms [1–3]. Microsporidia are also found in the form of spores with various diameters outside of the cell [4]. At present, approximately 187 genera and over 1300 species of Microsporidia have been described, among which almost half infect aquatic species and approximately 50 genera potentially infect aquatic arthropods [3, 5].

The greatest number of microsporidian species found in arthropods mostly infect insects, and only a few of these parasites have been detected in well-studied crustacean species [5]. Their widespread detection throughout the animal kingdom indicates the great evolutionary age of this group of parasites [1, 6].

Lake Baikal is the deepest and one of the oldest lakes in the world, and it has a rich endemic fauna with a predominance of arthropods [7]. Among the arthropods living in the lake, amphipods (Crustacea) are the most dominant group and are represented by more than 350 endemic species. Baikalian amphipods inhabit almost all depths and all types of substrates [8]. The age and geographical isolation of this group creates excellent opportunities for studying the diversity, evolution and genetics of host-parasite relationships. However, despite more than 150 years of study, data investigating the microsporidia of Lake Baikal remain incomplete. To date, only three studies have described species of microsporidia in this ecosystem [9]. The first discovery of Microsporidia in amphipods occurred in 1967: *Nosema kozhovi* in the Baikalian amphipod *Brandtia lata lata* [10]. The first molecular genetic studies of microsporidia were performed in the 21st century, with the first results obtained from a 2008 study of the diversity of microsporidia parasitizing the Baikalian amphipod *Gmelinoides fasciatus*. Six endemic microsporidia and one cosmopolitan species, *Dictyocoela duebenum*, were detected in this amphipod by SSU rDNA sequencing [11]. J.E. Smith and colleagues reported preliminary data regarding the discovery of 100 new species of microsporidia in 31 species of amphipods from Lake Baikal [12]. However, whether all of the species of Baikal amphipods were infected by microsporidia and the ratio of endemic to non-endemic species of microsporidia remain open questions.

It should be noted that in previous studies the microsporidian DNA was isolated from the entire body of Baikalian crustaceans [11]. Thus, data should be presented for both ecto- and endoparasite microsporidian species as well as contamination by microsporidian spores, which were accumulated in the digestion system. Microsporidia are found in the hemolymph of aquatic and terrestrial invertebrates in the form of spores and in hemocytes [13–16]. To avoid contamination (from digestive remains) and exosymbiotic microsporidia in the analyzed samples, the identification of species can be performed using the hemolymph. In the present study, we used molecular genetic techniques to detect microsporidia in the hemolymph of several endemic species of amphipods from Lake Baikal.

## Materials and Methods

### Sampling and location

Animals were collected from Lake Baikal close to the Bolshie Koty settlement (Irkutsk region, Eastern Siberia, Russia). No specific permissions were required for the samplings, locations or activities. The investigated amphipod species did not involve endangered or protected species.

**Fig 1:**
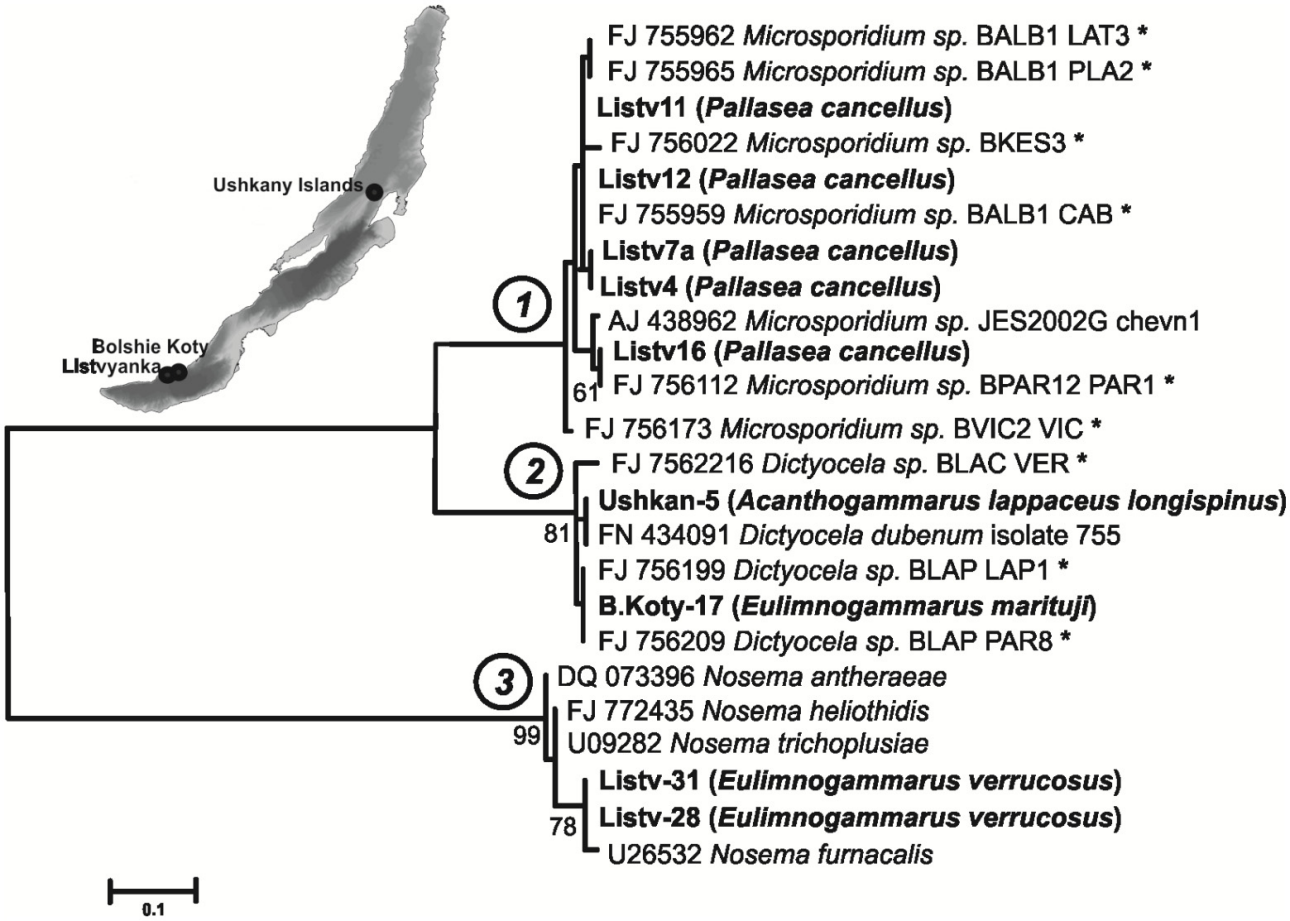
Maximum likelihood phylogenetic tree of Microsporidia. Bootstrap values (> 50%) are presented for nodes and branch lengths are drawn to scale. The species of microsporidia noted with ‘*’ found in Baikalian endemic amphipods (GenBank data).

Collections of littoral species of amphipods (*Pallasea cancellus* (Pallas, 1772), *Eulimnogammarus verrucosus* (Gerstf., 1858), *E*. *marituji* Baz., 1945) were performed on the southern shore of Lake Baikal (the village of Listvyanka: 51°50'58.67"N; 104°52'0.14"E and Bolshie Koty: 51°54'9.51"N; 105° 4'10.64"E) at a depth of 1 m using hand-nets. Deep-water species (*Acanthogammarus lappaceus longispinus* Tacht., 2000) were collected during a hydrobiological expedition in June 2014 (Ushkany Islands: N 53° 51^/^ 12^//^; E 108° 35^/^ 49^//^). No specific permissions were required for these locations and activities. Information regarding the collection points and the number of analyzed species is presented in Table 1 and Fig 1.

**Table 1:** The SSU rDNA sequences of microsporidia used for phylogenetic analysis.

Species	GenBank	Type of host	Location	References
*Microsporidium sp*. *PCN4*	KM977842	*Pallasea cancellus*	Listvyanka (Lake Baikal)	this paper
*Microsporidium sp*. *PCN7a*	KM977843	*Pallasea cancellus*	Listvyanka (Lake Baikal)	this paper
*Microsporidium sp*. *PCN11*	KM977844	*Pallasea cancellus*	Listvyanka (Lake Baikal)	this paper
*Microsporidium sp*. *PCN12*	KM977845	*Pallasea cancellus*	Listvyanka (Lake Baikal)	this paper
*Microsporidium sp*. *PCN16*	KM977846	*Pallasea cancellus*	Listvyanka (Lake Baikal)	this paper
*Dictyocoela sp*. *All*. *5*	KM977839	*Acanthogammarus lappaceus longispinus*	Ushkany Islands (Lake Baikal)	this paper
*Dictyocoela sp*. *BK17*	KP027301	*Eulimnogammarus marituji*	Bolshie Koty	this paper
*Nosema sp*. *Vr*.*28*	KM977840	*Eulimnogammarus verrucosus*	Listvyanka (Lake Baikal)	this paper
*Nosema sp*. *VR31*	KM977841	*Eulimnogammarus verrucosus*	Listvyanka (Lake Baikal)	this paper
*Dictyocoela sp*. *BLAC VER*	FJ756216	Eulimnogammarus verrucosus*	Lake Baikal	only GenBank
*Dictyocoela sp*. *BLAP LAP1*	FJ756199	Acanthogammarus lappaceus*	Lake Baikal	only GenBank
*Dictyocoela sp*. *BLAP PAR8*	FJ756209	Dorogostaiskia parasitica*	Lake Baikal	only GenBank
*Microsporidium sp*. *BALB1 LAT3*	FJ755962	Brandtia latissima latior*	Lake Baikal	only GenBank
*Microsporidium sp*. *BALB1 CAB*	FJ755959	Garjajewia cabanisii*	Lake Baikal	only GenBank
*Microsporidium sp*. *BPAR12 PAR1*	FJ756112	Dorogostaiskia parasitica*	Lake Baikal	only GenBank
*Microsporidium sp*. *BKES3*	FJ756022	Pallaseopsis kessleri*	Lake Baikal	only GenBank
*Microsporidium sp*. *BALB1 PLA2*	FJ755965	Micruropus platycercus*	Lake Baikal	only GenBank
*Microsporidium sp*. *BVIC2 VIC*	FJ756173	Acanthogammarus victorii*	Lake Baikal	only GenBank
*Dictyocoela duebenum isolate 775*	FN434091	*Gammarus duebeni duebeni*	Iceland: Atlantic Ocean	[28]
*Microsporidium sp*. *JES2002G isolate chevn1*	AJ438962	*Gammarus chevreuxi*	UK, River Avon	[24]
*Nosema furnacalis*	U26532	*Ostrinia furnacalis*	Asia (?)	[29]
*Nosema antheraeae*	DQ073396	*Antheraea pernyi*	China	[30]
*Nosema heliothidis*	FJ772435	*Helicoverpa armigera*	China	only GenBank
*Nosema trichoplusiae*	U09282	*Trichoplusia ni*	USA	[29]

‘*’—the species of microsporidia founds in endemic amphipod of Lake Baikal (GenBank data).

The identification of species and the hemolymph extraction were performed under laboratory conditions. Each sample (volumes ranging from 50–100 μl) contained a pool of hemolymph collected from several individuals of the same species. The number of individuals per sample depended on the size of the amphipods (2–5 individuals). Isolated samples were stored in liquid nitrogen.

### DNA isolation and PCR

Total DNA isolation from hemolymph was performed using the "Riboprep” reagent kit (AmpliSens, Russia). The concentration and purity of the isolated DNA was determined using the UV spectrophotometer UNICO 2802 (UNICO, USA).

The small subunit of ribosomal DNA (SSU rDNA) from the microsporidia was selected as a molecular phylogenetic marker. Amplification was performed in two stages (nested PCR) with universal primers for microsporidia: V1f: 5'- CACCAGGTTGATTCTGCCTGAC-3' [17]; 1342r: 5'- ACGGGCGGTGTGTACAAAGAACAG-3' [18]; 18sf: 5'- GTTGATTCTGCCTGACGT-3' [19]; and 981r: 5'- TGGTAAGCTGTCCCGCGTTGAGTC-3' [20]. Each PCR was performed in a Gradient Thermocycler (Biometra, Germany) in a volume of 25 μl and contained 10X PCR buffer, 2.5 mM dNTPs, 5 U/ml SynTaq DNA polymerase, 25 mM MgCl_2_, 10 pmol primers, and deionized water. The conditions for the first round were 95°C for 5 min, followed by 40 cycles of 95°C for 30 sec, 55°C for 30 sec, and 72°C for 90 sec, and then 72°C for 7 min. Conditions for the second round of PCR were 95°C for 5 min, followed by 35 cycles of 95°C for 30 sec, 52°C for 30 sec, and 72°C for 1 min, followed by 72°C for 7 min.

The PCR products of the second round were visualized in 1% agarose gels, and products of the expected size were excised from the gel and purified with ethanol and sodium acetate [21].

Sequencing of the amplified DNA fragments was performed using a Genetic Analyzer 3500 xL (Applied Biosystems) with the BigDye Terminator Cycle Sequencing kit v.3.1.

### Nucleotide sequence analysis

The sequences were aligned using the multisequence alignment program ClustalW within the BioEdit 7.0.5.3 environment [22]. The phylogenetic relationships between microsporidia representatives were determined using Mega 5.0 (Maximum Likelihood, HKY+G+I model). Bootstrap values were obtained for a consensus tree based on 1000 randomly generated trees using the same package [23].

## Results

Nine nucleotide sequences of SSU rDNA (738 bp) from three species of microsporidia were obtained from the hemolymph of all of the endemic amphipod species: *P*. *cancellus*, *E*. *verrucosus*, *E*. *marituji* and *A*. *lappaceus longispinus*. The dendrogram (Fig 1) was reconstructed using these and additional sequences from GenBank (see Table 1).

Four microsporidian nucleotide sequences were found in the *P*. *cancellus* hemolymph. These sequences showed a maximum similarity to the following species in GenBank: FJ755959 *Microsporidium sp*. BALB1 CAB, FJ756022 *Microsporidium sp*. BKES3, FJ755962 *Microsporidium sp*. BALB1 LAT, FJ755965 *Microsporidium sp*. BALB1 PLA2, FJ756112 *Microsporidium sp*. BALB1 PAR1, and FJ756173 *Microsporidium sp*. BVIC2 VIC CAB; these hosts are Baikalian endemic amphipods. The exception is AJ438962 *Microsporidium sp*. JES2002G chevn1, which was found in *Gammarus chevreuxi* from the Avon River, UK. All of these species are located in a single cluster in the dendrogram (marked as cluster 1).

The microsporidian nucleotide sequences were detected in the *Acanthogammarus lappaceus longispinus* and *Eulimnogammarus marituji* hemolymph samples and clustered with those of microsporidia of the genus *Dictyocoela* (marked as cluster 2). One species was identified with a close similarity to the following sequences from the Baikalian amphipods: FJ756316 *Dictyocoela sp*. BLAC VER, FJ756209 *Dictyocoela sp*. BLAC PAR8 and FJ756199 *Dictyocoela sp*. BLAC LAP1. The *Dictyocoela duebenum* isolate 775 (FN434091) was found in *Gammarus duebeni duebeni* (Iceland).

The two SSU rDNA sequences from the genus *Nosema* were detected in the hemolymph of *E*. *verrucosus* (marked as cluster 3). This is the first description of this species in an endemic crustacean of Lake Baikal.

Thus, the microsporidian DNA sequences that we detected in amphipods from Lake Baikal can be divided into three clusters: *Microsporidium*, *Dictyocoela* and *Nosema*.

## Discussion and Conclusion

This is the first study to demonstrate microsporidia in the hemolymph of Baikal endemic amphipods using molecular genetic methods. In all of the previous studies, microsporidian DNA was isolated from the entire body of the Baikalian crustaceans, thus providing data for both ecto- and endoparasite microsporidian species. The results of our study limited the number of microsporidia to endoparasite species only.

The microsporidian DNA detected in the *P*. *cancellus* hemolymph is similar to that of microsporidia found in some other endemic amphipods of Lake Baikal: *Garjajewia cabanisii* (FJ755959), *Pallaseopsis kessleri* (FJ756022), *Brandtia latissima latior* (FJ755962), *Micruropus platycercus* (FJ755965), *Dorogostaiskia parasitica* (FJ756112) and *Acanthogammarus victorii* (FJ756173). Therefore, one may assume that the majority of the detected species are likely to be endemic microsporidia. Therefore, one may assume that the majority of the detected species are likely to be endemic microsporidia excepting *Gammarus chevreuxi* (AJ438962) from the Avon River, UK. The nucleotide sequence of the SSU rDNA of this species is similar to those from several species of microsporidia found in fish [24].

It should be noted that the microsporidia of the genus *Dictyocoela* were found in two species of amphipods: *A*. *lappaceus longispinus* and *E*. *verrucosus* (GenBank data). It is known that some species of *Dictyocoela* stimulate feminization and change the sex ratio in their host populations [25]. There is hypothesis that this genus of microsporidia has a high degree of genetic diversity in populations of Eurasian amphipods and shows horizontal transmission [26].

Interestingly, we also detected some microsporidia that were similar to the genus *Nosema*. Many species in this genus primarily parasitize insects and belong to the class Terresporidia. According to Yu. S. Tokarev [27], “phylogenetic branching of the freshwater microsporidia among Terresporidia is indicative of multiple shifts of microsporidia between hosts of variable habitats, as well as of the secondary nature of adaptation of these species of parasites to the freshwater hosts.”

To summarize our data, we provide the first evidence that species of microsporidia belonging to three genera (*Microsporidium*, *Dictyocoela* and *Nosema*) reside in the hemolymph of Baikalian endemic amphipods. In the hemolymph of *E*. *verrucosus*, we detected the SSU rDNA of microsporidia belonging to the genus *Nosema*. In the hemolymph of *P*. *cancellus*, we found the DNA of *Microsporidium sp*. similar to that in other Baikalian endemic amphipods. *Dictyocoela sp*. was found in the hemolymph of *E*. *marituji* and *A*. *lappaceus longispinus*.
